# A Case Series of Rifabutin Use in Staphylococcal Prosthetic Infections

**DOI:** 10.1128/spectrum.00384-22

**Published:** 2022-05-11

**Authors:** Miranda Monk, Ramy Elshaboury, Alexander Tatara, Sandra Nelson, Monique R. Bidell

**Affiliations:** a Department of Pharmacy, Massachusetts General Hospital, Boston, Massachusetts, USA; b Department of Medicine, Division of Infectious Diseases, Massachusetts General Hospital, Boston, Massachusetts, USA; Emory University School of Medicine

**Keywords:** *Staphylococcus*, biofilms, drug interactions, prosthesis infections, rifabutin, rifampin

## Abstract

This case series describes seven patients who received rifabutin in place of rifampin combined with conventional antimicrobial therapy for treatment of hardware-associated staphylococcal infections. Infection recurrence, defined as need for unplanned surgical intervention within the evaluable follow up period after starting rifabutin, occurred in two patients. Two patients experienced possible treatment-associated adverse effects. Findings support future work to examine rifabutin use, when rifampin is not suitable, for adjunctive treatment of staphylococcal hardware infections.

**IMPORTANCE** This work evaluates real-world data and clinical outcomes when rifabutin is used in place of rifampin for adjunctive management of staphylococcal hardware-associated infections. This is the second case study looking at this specific use of rifabutin, signifying the current lack of clinical data in this area. Assessing use of rifabutin in this capacity is clinically important given its lower propensity for drug interactions compared to rifampin.

## INTRODUCTION

Bacterial biofilm formation is a challenging aspect of staphylococcal prosthetic hardware infections. Once implanted, host proteins such as fibrin and fibrinogen coat the surface of prosthetic material facilitating the binding of bacteria such as Staphylococcus aureus (1). Upon adherence, S. aureus synthesizes extracellular matrices composed of carbohydrates, proteins, and extracellular DNA. This biofilm limits conventional antimicrobial penetration to the site of infection, potentially leaving surviving organisms surrounding the prosthetic or implanted material that can contribute to infection relapse (2, 3). The rifamycins are a class of orally available agents that act by binding and inhibiting the β subunit of RNA polymerases among many types of bacteria with minimal effect on human RNA polymerases (4). Rifampin, a rifamycin, is commonly added to conventional antibiotic therapy for treatment of staphylococcal infections involving prosthetic material given that it (i) kills intracellular staphylococci, (ii) inhibits biofilm formation, and (iii) can assist in the penetration of traditional antimicrobials to the site of infection (5–7). However, use of rifampin can substantially complicate both infectious and noninfectious therapeutic management given its extensive drug-drug interaction profile (5). Rifampin exhibits potent induction of multiple cytochrome P450 (CYP450) enzymes and P-glycoprotein (P-gp) transport system proteins, which causes interactions with commonly prescribed medications that may warrant dose adjustment or switch to another therapeutic agent altogether during concomitant rifampin therapy (6, 8). Rifabutin, though used less commonly than rifampin in clinical settings, is another rifamycin that exhibits potent *in vitro* antistaphylococcal activity as well as fewer CYP450 enzyme interactions and no induction of P-gp proteins (5, 6, 9). Recently, Tuloup et al. studied a model-based analysis comparing rifampin and rifabutin drug-drug interaction profiles and concluded that interactions caused by rifampin were twice as potent as those caused by rifabutin (10). They further discussed that strong drug-drug interactions were unlikely when CYP substrates were coadministered with rifabutin dosed at 300 mg/day compared to rifampin at 600 mg/day. Though *in vitro* data suggest that rifabutin may be an effective and potentially safer alternative to rifampin with respect to drug-drug interactions, little clinical data exist to support use of rifabutin for treatment of biofilm-associated staphylococcal hardware infections. We therefore conducted a retrospective case series study examining outcomes of patients who received rifabutin in place of rifampin for adjunctive treatment of staphylococcal infections involving prosthetic material. Of note, this analysis focused mainly on clinical effectiveness of rifabutin, with a brief examination of potential rifabutin-associated adverse effects.

## RESULTS

Seven patients were included in the analysis, and case descriptions are featured in Table 1. All but one patient had methicillin-sensitive Staphylococcus aureus infection. The remaining patient had Staphylococcus epidermidis and Cutibacterium acnes as recovered pathogens. All patients underwent surgical intervention. Three patients had hardware retained, two patients had hardware placed into an infected bed, one patient had a hardware revision, and one patient underwent left-ventricular assistance device (LVAD) removal followed by heart transplant. Patients were treated with cefazolin (*n* = 4), oxacillin (*n* = 1), vancomycin (*n* = 1), or cefepime (*n* = 1) in combination with rifabutin as initial therapy. Rifabutin, in combination with primary antibiotic therapy, was chosen over rifampin in all scenarios out of concern for significant drug-drug interactions with chronic home medications. Rifabutin was selected over rifampin due to anticipated interactions with psychiatric medications (*n* = 5), methadone (*n* = 3), direct-acting oral anticoagulants (*n* = 2), blood pressure/cholesterol medications (*n* = 2), and antiepileptic medications (*n* = 3). Rifabutin 300 mg daily, except patient 4 who received 450 mg daily, was started within 4 to 7 days of surgical intervention in all cases. Doses were selected on a case-by-case basis at the discretion of the treating Infectious Diseases physician and Infectious Diseases pharmacist. In all cases, rifampin susceptibility was used as a proxy for rifabutin susceptibility when data were available (rifampin MICs of ≤0.5 mg/L for all except patients 4 and 6, for which MICs were unavailable).

**TABLE 1:** Case summaries of patients who received rifabutin for staphylococcal prosthetic infections^*a*^

Case summary no.	Infection	Planned rifabutin duration	Rifabutin length of therapy	Reason for rifabutin/concurrent interacting medications	Microbiology	Intravenous antimicrobial therapy	Oral maintenance	Surgical management	Adverse effects	Clinical outcome
1	Prosthetic joint infection (PJI)	3 months	3 months	Methadone, rivaroxaban, lamotrigine	MSSA	Oxacillin × 6 weeks	Doxycycline × 12 months	I&D, liner exchange, hardware retained	None	No recurrence at 18 months
2	PJI/skin and soft tissue infection	12 weeks	10 weeks	Bupropion, cariprazine, vortioxetine	MSSA	Cefazolin × 6 weeks	Trimethoprim-sulfamethoxazole × 6 weeks	I&D, hardware retained	Nausea	No recurrence at 12 months
3	Spinal fusion hardware infection	5 weeks	5 weeks	Methadone	*S. epidermidis*, *C. acnes*	Vancomycin × 2 weeks	Doxycycline × 4 wks	Stage back-front-back hardware revision with 3 column osteotomies	None	No recurrence at 18 months
4	Osteomyelitis/discitis/epidural abscess	6 weeks	11 weeks	Methadone	MSSA, *K. pneumoniae*	Cefepime then meropenem × 7 weeks	Cefadroxil × 12 months (planned)	I&D, laminectomy, disc fusion, hardware retained	None	Persistent thoracic infection, requiring one washout at 7 weeks, no further recurrence at 15 months
5	Left-ventricular assistance device (LVAD) infection	6 weeks	7.5 weeks	Lamotrigine, lurasidone, warfarin	MSSA	Cefazolin × 11 weeks (until heart transplant)	NA	Heart transplant and LVAD removal	None	No recurrence at 40 months
6	Spinal fusion hardware infection	8 weeks	4 weeks, followed by 3 months	Apixaban, atorvastatin, oxycodone	MSSA	Cefazolin then daptomycin × 8 weeks	Doxycycline × 12 months	I&D, decompression, disc fusion, hardware inserted into infected bed	Leukopenia	No recurrence at 22 months
7	Spinal fusion hardware infection	6 weeks	6 weeks	Metoprolol, oxycodone, rosuvastatin, amlodipine	MSSA	Cefazolin × 6 weeks	Doxycycline (duration unknown)	I&D, hardware into infected bed, disc fusion	None	Required I&D at 8 weeks, determined to be noninfectious, no further recurrence at 13 months

a MSSA, methicillin-sensitive Staphylococcus aureus; I&D, incision and drainage; NA, not applicable.

Planned duration of rifabutin ranged from 5 to 12 weeks. Patient 5, who had LVAD removal and heart transplantation, received rifabutin for a portion of the intravenous therapy course and did not require oral maintenance therapy. All other patients remained on rifabutin throughout their entire course of intravenous primary therapy and were started on oral maintenance therapy thereafter. Five of seven patients continued rifabutin for a portion of their oral maintenance therapy.

Two patient cases met the definition of infection recurrence. Patient 4 presented with a persistent paraspinal phlegmon and edema involving thoracic hardware. The patient was felt to have recurrence upon surgical washout resulting in change of antibiotic therapy, but cultures remained negative and hardware was retained. Of note, change in antibiotic therapy was also supported by concerns for antibiotic-associated vomiting and renal impairment. No further surgical interventions were noted after this change in therapy, and the patient was recurrence-free at 15 months. Patient 7 was noted to have a fluid collection at the surgical site and was taken for surgical drainage 8 weeks after starting rifabutin therapy. However, this collection was deemed to be noninfectious as no inflammatory markers or signs of infection were noted, no purulence was observed during surgical intervention, and operative cultures were negative. This patient transferred to another facility after starting oral maintenance with doxycycline but did not experience further recurrence when evaluated 13 months after starting rifabutin.

Overall, rifabutin was well tolerated; however, two patients required holding therapy due to possible adverse effects. One patient experienced leukopenia and elevated serum creatinine approximately 1 month into a planned 8-week course of cefazolin and rifabutin. After a 4-week rifabutin holiday and transition of cefazolin to daptomycin, rifabutin was resumed with no subsequent adverse effects. Another patient had possible rifabutin-associated nausea that resulted in a week-long pause with no further intolerance upon reintroduction for the remaining course. While a comprehensive safety analysis was outside the scope of this study, a brief chart review indicated that no readily identifiable major sentinel events (i.e., reports of seizure, embolic events) occurred that could be attributable to a clinically relevant interaction with rifabutin and select concomitant high-risk medications.

## DISCUSSION

This case series suggests that rifabutin can be considered an alternative adjunctive treatment of staphylococcal infections complicated by biofilm production when drug-drug interactions prohibit safe use of rifampin. Although two patients met our definition of infection recurrence, surgical cultures in both cases were negative for any organism growth, suggesting either that the underlying process was noninfectious or that the associated bacterial burden was low (e.g., due to being on antibiotics at the time). Few adverse events were observed with use of rifabutin, and none recurred on drug rechallenge. While we did not formally evaluate rifabutin-associated drug interactions, a brief chart review suggested that no sentinel events occurred as a result of clinically significant drug-drug interactions. To our knowledge, this is the second real-world case series assessing outcomes associated with use of rifabutin for staphylococcal biofilm infections. Our findings are similar to those described by Doub et al., who noted no infection recurrence among 10 patients who received rifabutin in place of rifampin for prosthetic joint infections due to drug-drug interaction concerns (11). Rifabutin was well tolerated without side effects in their study.

While collective findings are promising related to use of rifabutin in place of rifampin, there are notable considerations when applying our findings, aside from the inherent limitations associated with a small retrospective case series analysis. Rifabutin, at least theoretically, may not be a suitable alternative to rifampin for all prosthetic or biofilm-associated infections given the pharmacokinetic differences between these agents. Rifabutin is known to have considerably higher tissue concentrations than plasma concentrations, whereas rifampin is equally concentrated in the tissue and plasma (4). Therefore, rifampin may be preferred in infections in which sufficient plasma concentrations are desired. We also did not closely examine the impact of rifabutin selection on drug-drug interactions or specific safety findings related to this or the potential clinical impact of using different doses of rifabutin. While the metabolic induction potential with rifabutin is considered to be lower than that of rifampin, rifabutin itself is a substrate of CYP3A4 and, therefore, is subject to increased concentrations when combined with CYP3A4 inhibitors such as fluconazole or clarithromycin (12). This reinforces the need to evaluate all potential drug-drug interactions with use of rifabutin in consultation with medication databases and/or clinical pharmacists.

In summary, the limited experience published to date suggests that rifabutin is well tolerated and may be considered a potential alternative to rifampin for staphylococcal biofilm infections in patients who have complicated medication regimens subject to CYP450 and P-gp drug-drug interactions. However, these findings are still largely hypothesis-generating. More clinical and prospective data are necessary in order to substantiate rifabutin as an appropriate therapeutic alternative to rifampin for adjunctive treatment of staphylococcal prosthetic infections.

## MATERIALS AND METHODS

All patients at Massachusetts General Hospital who received rifabutin in place of rifampin for staphylococcal hardware infections between January 2018 and March 2021 were eligible for inclusion. Patients were included if they reported taking >50% of their prescribed doses, as documented in the medical record. The primary outcome of the study was infection recurrence, defined as unplanned need for surgical intervention from the point of starting rifabutin until the latest date with evaluable data. Rifabutin-associated adverse effects, planned duration of treatment, and concomitant medication use leading to selection of rifabutin over rifampin were also evaluated.

## References

[1] Hiltunen AK, Savijoki K, Nyman TA, Miettinen I, Ihalainen P, Peltonen J, Fallarero A. 2019. Structural and functional dynamics of *Staphylococcus aureus biofilms* and biofilm matrix proteins on different clinical materials. Microorganisms 7:584. doi:10.3390/microorganisms7120584. 31756969 PMC6955704

[2] Karygianni L, Ren Z, Koo H, Thurnheer T. 2020. Biofilm matrixome: extracellular components in structured microbial communities. Trends Microbiol 28:668–681. doi:10.1016/j.tim.2020.03.016. 32663461

[3] Sanchez CJ, Shiels SM, Tennent DJ, Hardy SK, Murray CK, Wenke JC. 2015. Rifamycin derivatives are effective against staphylococcal biofilms in vitro and elutable from PMMA. Clin Orthop Relat Res 473:2874–2884. doi:10.1007/s11999-015-4300-3. 25896136 PMC4523531

[4] Rothstein DM. 2016. Rifamycins, alone and in combination. Cold Spring Harb Perspect Med 6:a027011. doi:10.1101/cshperspect.a027011. 27270559 PMC4930915

[5] Albano M, Karau MJ, Greenwood-Quaintance KE, Osmon DR, Oravec CP, Berry DJ, Abdel MP, Patel R. 2019. In vitro activity of rifampin, rifabutin, rifapentine, and rifaximin against planktonic and biofilm states of staphylococci isolated from periprosthetic joint infection. Antimicrob Agents Chemother 63. doi:10.1128/AAC.00959-19. PMC6811393 31451499

[6] Chambers HF. 2020. Rifabutin to the rescue? J Infect Dis 222:1422–1424. doi:10.1093/infdis/jiaa403. 32914842

[7] Zimmerli W, Sendi P. 2019. Role of rifampin against staphylococcal biofilm infections in vitro, in animal models, and in orthopedic-device-related infections. Antimicrob Agents Chemother 63. doi:10.1128/AAC.01746-18. PMC6355554 30455229

[8] Niemi M, Backman JT, Fromm MF, Neuvonen PJ, Kivistö KT. 2003. Pharmacokinetic interactions with rifampicin: clinical relevance. Clin Pharmacokinet 42:819–850. doi:10.2165/00003088-200342090-00003. 12882588

[9] Karau MJ, Schmidt-Malan SM, Albano M, Mandrekar JN, Rivera CG, Osmon DR, Oravec CP, Berry DJ, Abdel MP, Patel R. 2020. Novel use of rifabutin and rifapentine to treat methicillin-resistant Staphylococcus aureus in a rat model of foreign body osteomyelitis. J Infect Dis 222:1498–1504. doi:10.1093/infdis/jiaa401. 32914837

[10] Tuloup V, France M, Garreau R, Bleyzac N, Bourguignon L, Tod M, Goutelle S. 2021. Model-based comparative analysis of rifampicin and rifabutin drug-drug interaction profile. Antimicrob Agents Chemother 65. doi:10.1128/AAC.01043-21. PMC8370242 34228545

[11] Doub JB, Heil EL, Ntem-Mensah A, Neeley R, Ching PR. 2020. Rifabutin use in staphylococcus biofilm infections: a case series. Antibiotics 9:326. doi:10.3390/antibiotics9060326. 32545793 PMC7345564

[12] Jordan MK, Polis MA, Kelly G, Narang PK, Masur H, Piscitelli SC. 2000. Effects of fluconazole and clarithromycin on rifabutin and 25-O-desacetylrifabutin pharmacokinetics. Antimicrob Agents Chemother 44:2170–2172. doi:10.1128/AAC.44.8.2170-2172.2000. 10898693 PMC90031

